# Physician drug dispensing in Switzerland: association on health care expenditures and utilization

**DOI:** 10.1186/s12913-016-1470-y

**Published:** 2016-07-08

**Authors:** Maria Trottmann, Mathias Frueh, Harry Telser, Oliver Reich

**Affiliations:** Polynomics, Baslerstrasse 44, Olten, Switzerland; Department of Health Sciences, Helsana Group, Zurich, Switzerland

**Keywords:** Prescription drugs, Physician dispensing, Health care expenditures

## Abstract

**Background:**

Several countries recently reassessed the roles of drug prescribing and dispensing, either by enlarging pharmacists’ rights to prescribe (e.g. the US and the United Kingdom) or by limiting physicians’ rights to dispense (e.g. Taiwan and South Korea). While integrating the two roles might increase supply and be convenient for patients, concern is that drug mark-ups incite providers to prescribe unnecessary drugs. We aimed to assess the association of physician dispensing (PD) in Switzerland on various outcomes.

**Methods:**

We performed a retrospective cohort study, using health care claims data for patients in the year 2013. The analysis of the association of PD was perfomed using a large patient level dataset and several target variables, including the number of different chemical agents, share of generic drugs, number of visits to physicians and expenditures. Different multivariate econometric models were applied in order to capture the association PD on the target variables.

**Results:**

A total of 101’784 patients were enrolled in 2013, whereas 54 % were PD patients. We find that PD is associated with lower pharmaceutical expenditure per patient, which can be explained by an increased use of generic drugs. The decrease is compensated by higher use of physician services. We find no significant impact of physician dispensing on total health care expenditure.

**Conclusions:**

Our study offers insights for policy makers who are (re-)considering the separation between drug prescribing and dispensing, either by allowing physicians to dispense or pharmacists to prescribe certain drugs. In terms of total health care expenditures, we find no difference between the two systems, so we are doubtful that changing dispensing rights are a good measure to contain cost, at least in Switzerland.

## Background

Access to high quality drug prescriptions and drug supply are major issues in ambulatory care. In this context, many countries have recently reassessed the roles of pharmacists and physicians. Countries such as the US, Canada, and the United Kingdom, who face a shortage of primary care providers, have enlarged pharmacist’s rights to dispense, effectively integrating the two roles (Latter et al. [1], Abramowitz et al. [2]). While integration might be convenient for patients, concern is that drug mark-ups raise well known problems such as provider-induced-demand (PID) (e.g. Arrow [3]).

In South East Asia, the two roles were traditionally integrated as physicians prescribed and dispensed drugs. Recent policy reforms in many countries (e.g. South Korea, Taiwan, Japan and China) aimed at a stricter separation (Eggleston [4]). A number of empirical studies support the notion that PID is a problem. For example, Iizuka et al. [5] find that mark-ups significantly influence the choice of anti-hypertensive drugs by Japanese physicians and using the cheapest drugs could markedly reduce drug expenditures. Chou et al. [6] find that Taiwanese physicians who no longer earned drug mark-ups decreased both the frequency of a prescription and the prescribed volume.

The Swiss experience with prescribing and dispensing is quite unique. Drug dispensing is regulated on the cantonal (‘state’) level and some parts of the country have a long tradition with physician dispensing (PD) while other cantons strictly separate the two roles. In the cantons that allow dispensing, physicians gain considerable income from this activity. Research from Hunkeler [7] estimates that dispensing primary care physicians on average earn 28 % of their income from drug mark-ups. For dispensing specialists, 7 % of earnings come from dispensing.

The Swiss empirical literature focused on the effects of PD on health care expenditure and reports surprisingly contradictory findings. Several studies have analyzed physician dispensing using aggregate cantonal data, which has the disadvantage that sample sizes are small (there are only 26 cantons). Beck et al. [8] relate per-capita drug expenditure to physician dispensing regulation and other characteristics of cantons and find that PD is associated with a strong increase in drug expenditures. In their data, pharmaceutical expenditures are assigned to the canton of the provider’s location and divided by the cantonal population to calculate average cost per head. There is a substantial outlier in this data because a small canton («Thurgau») hosts a large mail order drug store serving many non-local patients. Because Thurgau is a PD canton and the sample size for regression is small, this outlier might influence the coefficient of PD upwardly. Using a panel data approach, Reich et al. [9] find a small positive effect of PD on health care expenditures (HCE). A different result was obtained by an earlier study perfomed by Vatter et al. [10], who identify a significantly negative effect of the share of PD providers on per capita HCE. More surprisingly still, Schleiniger et al. [11] estimate a significantly negative effect of PD on drug expenditure. Their data is similar to that of Beck et al. [8], but expenditures are assigned to patient location instead of provider location. Still, the small sample size might mean that regression results are influenced by outliers.

Other studies used physician level data to analyze the effects of dispensing. The results estimated by Busato et al. [12] illustrate that PD is associated with lower physician expenditure in primary care and some (but not all) specialties. Focusing on dispensing general practitioners (GP) and specialist physicians, two recent studies by Kaiser et al. [13] (only specialist physicians) and Burkhard et al. [14] find that dispensing physicians have markedly higher drug cost per patient than their non-dispensing colleagues. With data on the physician level, the implication of these results on total health care expenditure remain unclear. Indeed, patients who see many physicians have lower expenditures *per physician* than a loyal patient who see only one physician. In addition, it is challenging to control for practice size in these studies. Physicians with large patient panels may play a strong role in the provision of services for their communities and therefore prescribe more, regardless of the PD status (Kaiser et al. [13] don’t control for this, Burkhard et al. [14] don’t report whether they do).

Rischatsch et al. [15] analyzed the choice between generic and brand-name drugs and find that generic substitution is more common in the PD market than in the pharmacy based system. Exploiting the small area variation in Switzerland, Fillipini et al. [16] find that PD is associated with slightly higher use of antibiotic drugs. Finally, Blozik et al. [17] looked at the frequency of potentially inappropriate medications (PIM) among elderly patients and find that it is more frequent in the PD than in the pharmacy sector.

As there is no consensus in the literature about the size or even the sign of the association between PD and health care expenditure in Switzerland, further research on the topic is necessary. This study sheds new light on the issue by analyzing individual patient data from the canton of Zurich, which is the most inhabited canton in Switzerland. We expect dispensing rights to influence drug prescribing behaviour and the supply of other physician services, so we analyze a set of different target variables, such as drug quantities, share of generic drugs, number of visit to physicians and physician expenditures. In addition, we analyze the impact of PD on total health care expenditure, which is of considerable interest for health policy. Using patient level data allows us not only to control for a wide range of individual characteristics but also to follow the patient through different parts of the health care system, assessing the total amount of resources used for her treatment.

The remainder of this paper is structured as follows. Section 2 contains a description of the policy setting and ambulatory care in Switzerland. In section 3, we formulate hypotheses on the influence of PD on physician behaviour and derive a testing strategy. The data and the econometric specifications are described in section 4. Section 5 discusses estimation results while section 6 rounds off with a summary and conclusions.

## Policy setting

### Ambulatory care in Switzerland

Ambulatory care in Switzerland is mostly provided by private for-profit physicians and pharmacists. Their services are mainly funded by mandatory insurance, with patients paying deductibles and cost-sharing up to a maximum per year. Patients can choose freely among all licensed physicians and pharmacists. Insurers are obliged to reimburse consultation fees to physicians according to a nationwide fee-for-service schedule that is collectively bargained between the providers’ and the insurers’ association. Pharmacists receive consultations fees for advising patients on their drug therapy. In addition, pharmacists and dispensing physicians earn drug mark-ups. Drug prices are regulated by the Federal Office for Public Health and patients (or their insurers) generally pay the same price whether they buy from physicians or at pharmacies. Mark-ups are fixed as the difference between the regulated ex-factory price and the consumer price. Still, there is room for negotiations on drug mark-ups as physicians and pharmacists get rebates on regulated ex-factory prices. Rebates are more common in the generic market, because different producers compete for market share.

### Physician dispensing (PD)

PD is regulated at the cantonal (“state”) level, and a variety of regulations can be observed within Switzerland (see [13] for a review). In French and Italian speaking regions, PD is unknown except in emergencies and for special treatment options. In the German speaking regions, only one canton (Basel-City) has such a rigid regulation. Many of the remaining cantons allow physicians to dispense on their own account, while some others apply mixed systems. In all the cantons that allow physician dispensing, patients have a right to ask physicians for written prescriptions and fill them at the pharmacy of their choice. Several cantons oblige dispensing physicians to explicitly inform patients about this right.

## Hypotheses and testing strategy

### Hypotheses

In this section, we formulate hypotheses about how PD might influence the decision making of physicians. The first aspect that comes to mind is drug quantities. Dispensing physicians earn positive drug mark-ups, so their prescription decisions can be seen as «self-referrals», a term used to describe referrals to resources (partly) owned by the treating physician (Mitchell and Scott [18]). The theory of «physician induced demand» (PID), originally formulated by Evans [19], states that physicians might tilt their patients’ demand curve towards services that lie in their own interest. Physicians are able to do this because they know much more about the possibilities and consequences of treatment and often take medical decisions on the patients’ behalf (physician agency, Arrow [3]). It is important to note that demand inducement only occurs if the physician advises services against his or her own interpretation of the patients’ best interest. Shifting demand towards the patients’ optimum, the physician does his or her job as the patient’s perfect agent. Although the PID-hypothesis is debated in the health economic literature (Feldman and Sloan [20]), there is a substantial body of literature which shows that physician ownership influences medical decisions (see Johnson [21] for a review). Building on this line of research, we test the hypothesis that PD leads to increased drug quantities. The second aspect is drug prices. The prices that patients or their insurers pay are regulated to be equal at pharmacists or physicians for the same product. Still, differences occur if there are different treatment options available – for example brand-name and generic drugs. The study of Rischatsch et al. [15] found that dispensing physicians dispense more (cheaper) generic drugs than pharmacies do. The authors offer two explanations for this: First, dispensing physicians are likely to be better informed about drug prices than non-dispensing physicians and might prescribe more generics to save the patient money. Second, dispensing physicians are likely to earn higher mark-ups for generic drugs because of generous rebates on ex-factory prices. With high competition in the generic market, producers give these rebates in order to gain market share. We believe that both of the factors reported by Rischatsch et al. [15] could influence prescribing decisions and test the hypothesis that physician dispensing leads to lower drug prices.

For consultation services, physicians are remunerated by fee-for-service (see also «policy setting»). PD can be seen as an increase in the fee per visit because physicians earn additional drug mark-ups (bearing in mind that drug prescriptions are frequent during outpatient visits). In the health economics literature, the effects of a fee increase on quantities is debated. Standard microeconomic theory predicts that producers increase supply when (relative) prices are high, and there is evdience that this applies to outpatient medical care (Clemens and Gottlieb [22]). However, the «target income hypothesis» of physician behaviour predicts that *lower* fees lead to more services, at least in the short run when the number of suppliers is constant (see e.g. Nguyen and Derrick [23]). Because physicians in Switzerland have other (profitable) options than providing outpatient consultations and PD has been known for a long time, we believe that the former argument is more convincing and test the hypothesis that PD increases the supply of physician services, especially in primary care where the additional income from PD is substantial [7]. We also believe that physicians are in a strong position to influence demand. For example, dispensing physicians might write less long-term prescriptions than other physicians. Long-term prescriptions are usually filled by pharmacies, leading to forgone income from drug dispensing. In this case, PD-patients on long-term drug therapy will need to visit physicians more often. Table 1 illustrates the study hypotheses.

**Table 1:** Overview of hypotheses

Hypothesis 1:	PD increases drug quantities
Hypothesis 2:	PD increases the use of generic drugs, resulting in lower drug prices.
Hypothesis 3:	PD increases the supply of physician consultations, notably by primary care physicians

### Testing strategy

In order to tests these hypotheses, we analyze a set of different target variables (see Table 2). To approximate drug quantities (hypothesis 1), we analyze the number of different active agents that were dispensed to patients during the observation period. As an indicator of drug prices, we analyze a [0/1]-indicator whether the patient used generic drugs for more than 50 % of her drug expenditures. We expect a positive influence of PD (hypothesis 2). In addition, we also analyze the effects of PD on drug expenditure per patient. As PD is expected to increase drug quantities and lower drug prices, we do not have a clear expectation on how PD should affect drug expenditures.

**Table 2:** Overview of target variables and expected influence of physician dispensing (PD)

Variable	Expected sign of PD-variable
Number of different active agents per patient	+
Share of generic drugs	+
Drug expenditure	unclear
Number of visits to general practitioner	+
Number of visits to specialists	+
Physician expenditure	+
Total health care expenditure	unclear

Turning now to physician services, hypothesis 3 states PD is associated with more consultations per patient. We expect this increase to be stronger in primary care than in specialist care, because drug dispensing is more influential on the income of primary care physicians. As the number of consultations increases, we also expect an increase in the expenditures for physician services.

We also analyze the effect of PD on total health care expenditure, because this variable is of considerable policy interest. We have no clear hypothesis on the influence of PD.

## Methods

### Modelling physician dispensing

As not all physicians legally entitled to dispense choose to do so, legal status is a poor measure of dispensing activities in the market. Using a data-driven approach instead, we define a physician as dispensing when the following two conditions are fullfilled: First, the physician prescribed drugs worth more than Swiss Francs (CHF) 12,500 in the 6-month observation period. Second, the physician dispensed more than half of the drugs he or she prescribed. This definition was used in previous work by Helsana insurance company and Hunkeler [7].

**Fig. 1:**
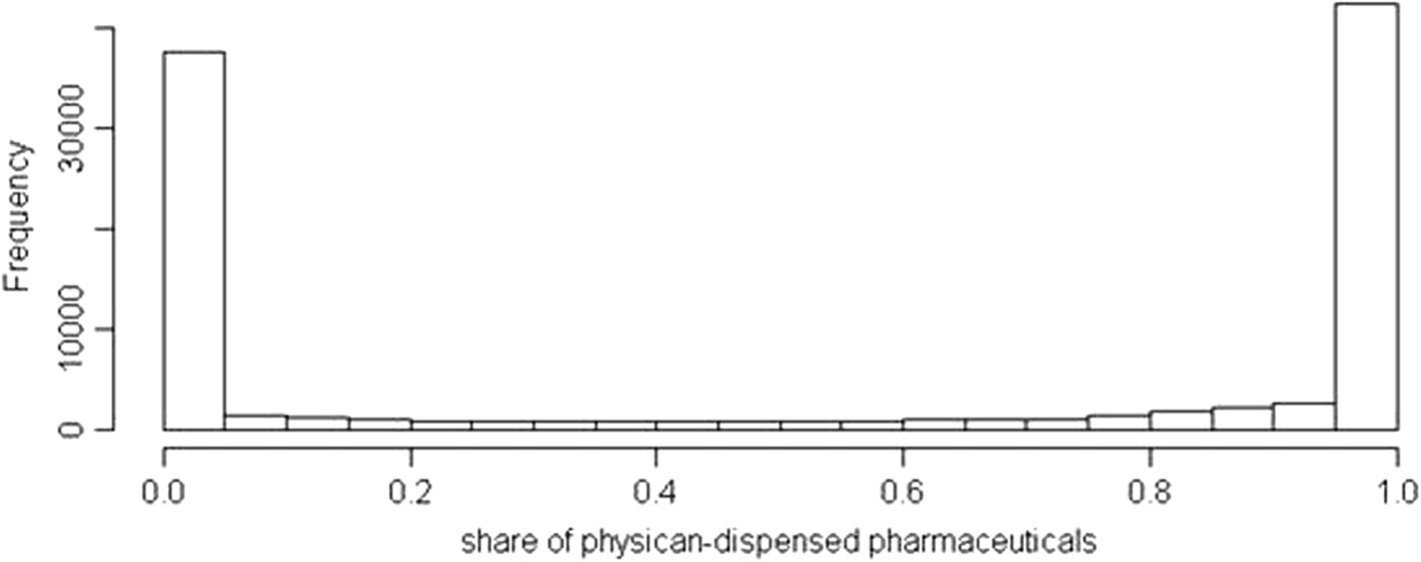
(Bimodal) Distribution of physician-dispensed drug expenditure per patient

For the analysis of patient level data, we define a patient as PD-patient if he or she received 50 or more percent of his or her drugs (measured by pharmaceutical expenditure turnover) from a dispensing physician. For most of the patients in the dataset, the share of physician-dispensed pharmaceuticals was either very high, or very low, so the choice of cut-off point does not influence the results (see the distribution in Fig. 1).

### Data and descriptive statistics

We use a large dataset of individual claims from the year 2013 covering the period 1.1.2013 to 30.6.2013, which was provided by Helsana, a large health insurance company. Claims files contained information on the enrollees’ age, sex, health insurance plan, health expenditure and utilization, and prescribed drugs including the ingredients as defined by the Anatomical Therapeutic Chemical (ATC) code from the WHO. The pharmaceutical expenditures are either induced directly from the dispensing physician or indirectly from prescriptions filled in community pharmacies. Several restrictions were made to the data: First, we excluded enrollees with no pharmaceutical spending during the observation period on the grounds that we cannot distinguish PD and non-PD patients if no drugs were bought. Second, we excluded patients who received more than 20 % of their prescriptions from physicians working in hospitals. Although hospitals are important agents in the health care market, they are not in the focus of our study. Third, we also excluded children under the age of 18 years, because physicians always dispense some of the children’s medicines (such as vaccines), which could confound the effects of PD.

Descriptive statistics are shown in Table 3. The dataset includes 101,784 patients, 54 % of which are PD patients. PD patients are slightly older on average than non-PD patients. The share of people with high deductibles is slightly lower among PD than among non-PD patients. Still, PD patients had lower total health care cost on average in the previous year.

**Table 3:** Characteristics patients in physician dispensing (PD) group versus non-PD group, year 2013

	Non-PD	PD	*p*-value
N	46,445	55,339	
Control variables, observed on 1.1.2013 [means and standard deviation or in %]			
Age	55.4 (19.02)	57.6 (19.37)	0.0***
Share of females	0.61 %	0.57 %	0.0***
Share of high deductibles	0.22 %	0.21 %	0.0***
Standard contract (non-managed care)	0.59 %	0.57 %	0.0***
Health care expenditure in previous year in Swiss francs	5,204 (8,516)	4,818 (8,704)	0.0***
Agricultural-mixed municipalities	0.4 %	1.2 %	0.0***
Agricultural municipalities	0.0 %	0.0 %	0.0***
Affluent municipalities	7.8 %	10.2 %	0.0***
Touristic municipalities	0.2 %	0.1 %	0.0***
Industrial and tertiary municipalities	1.3 %	3.1 %	0.0***
Rural commuting municipalities	1.0 %	2.5 %	0.0***
Periurban municipalities	5.5 %	14.0 %	0.0***
Suburban municipalities	25.4 %	53.6 %	0.0***
Urban centers	58.5 %	15.2 %	0.0***
Target variables, observed 1.1.2013 - 30.06.2013			
Pharmaceutical expenditure per patient in Swiss francs	538 (1,299)	469 (1,031)	0.0***
Share of generic drugs	19.3 %	24.6 %	0.0***
Number of different active agents per patient	5.40	5.68	0.0***
Number of GP visits per patient	2.73	3.13	0.0***
Number of specialist visits per patient	2.80	2.13	0.0***
Physician expenditure per patient in Swiss francs	816 (1,217)	753 (1,513)	0.0***
Health care expenditure per patient in Swiss francs	2,559 (4,536)	2,406 (4,876)	0.0***

Source: Claims data from Helsana insurance company, own calculations, Standard deviations in parentheses

**Table 4:** Target variables and model specifications

Variable	Type	Specification
No. of different active agents	count data	negative binominal
Share of generic drugs	binominal variable	logit
Drug cost	continuous	glm, log link, gamma variance
Physician expenditures	continuous, peak at zero	two-part model
No. of visits gp	count	negative binominal
No. of visits specialists	count	negative binominal
Total health care cost	continuous	glm, log link, gamma variance

Control variables: Age, gender, deductibles, expenditure in previous year, number of hospital visits/stays, 22 pharmaceutical cost groups to indicate illness

The strongest difference between PD and non-PD patients is found regarding the type of municipalities. Non-PD patients often live in urban centres, while PD patients are more likely to be in suburban municipalities. Still, it is important to note that the whole canton of Zurich is quite densely populated and access to medical services is high everywhere.

The target variables are shown in the bottom third of Table 3. For target variables like pharmaceutical expenditures and total health care expenditures, we observe very high standard errors, which is typically observed with health data. PD patients have lower pharmaceutical expenditure on average than non-PD patients, and a higher share of generic drugs. PD patients have a higher number of consultations by GPs but a lower number of visits to specialists than non-PD patients.

### Econometric methods

In this study we aim to compare patients that receive drugs predominately from physicians to patients who receive drugs at the pharmacy. Using a comprehensive patient-level dataset, we are confident that we are able to control for many relevant differences in patient populations. Variables potentially related to the choice of the physician and therefore the dispensing status could not be included in the analysis due to data availability. On the physician side, we control for specialty only because more detailed data on physicians was not available. Hence, this study cannot investigate the determinants driving the dispensing choice of the physician. This part is unique and ought to be considered in further research.

The different target variables discussed in section 3 call for different econometric specifications (see Table 4). The [0/1]-indicator variable of drug prices (more than 50 % of generics) was estimated by a logit model. For the continuous, non-negative numbers like drug cost and total cost, we used a glm specification with a log link and a gamma-type variance structure. The log-link was chosen because the distribution of this variable is highly skewed to the right. The Park test suggested by Manning et al. [24] indicated that even on the log scale, the error variance grew proportionally with the fitted values, so we used the ‘gamma family’ specification.

The cost for physician services are a continuous variable as well, but have a high share of zero values (about 10 %). Therefore, we use the two-part model proposed by Duan et al. [25]. On the first part, the probability of using any care is estimated by a probit model. On the second stage, the cost for patients with positive cost is estimated by a glm specification with a log link and a gamma-type variance structure.

Last but not least, variables like the number of physician visits or the number of different active agents take on non-negative discrete values (including zero). This calls for a specification using a ‘count data’ model like poisson or negative binominal regression. In order to choose between these models, we apply the test for over-dispersion suggested by Cameron et al. ([26], p. 671). The result shows that overdispersion is a problem and therefore we choose the negative binomial specification.

In non-linear models such as logit or two-part models, the marginal effect of an independent variable on the outcome variable depends on the values of all control variables Norton et al. [27]. One could calculate the marginal effects at a specific point of the distribution, for example at sample means. In our case, most of the control variables are categorical, and some of them are strongly correlated, so sample means might give misleading results. We therefore assess the marginal effects for each individual and average them. The individual marginal effects are calculated by estimating the fitted values twice, first while assuming that all patients were PD patients, second while assuming all patients where non-PD patients. The sample average marginal effect was calculated. Standard errors were obtained by bootstrapping, ie drawing with replacement 1000 samples of equal size (50,000 patients).

## Results and discussion

### Drugs

Hypothesis 1 states that PD is associated with increased drug quantities. As shown in Table 5 (column 1), this seems to be the case for the number of active agents per patient. The coefficient of the PD variable is significantly positive and indicates that PD leads to a 2.5 % increase in the number of different active agents. The mean marginal effect amounts to 0.13 units per patient, meaning that roughly one in eight PD patients on average is prescribed an additional chemical agent. This finding is in line with previous research [16] which reported higher drug quantities in areas with many PD physicians.

**Table 5:** Results of the multivariate regression analysis of the impact of physician dispensing (PD) on drug utilization

Target variable	Number of different active agents	Share of generics	Pharmaceutical expenditure	Pharmaceutical exp. and pharmacists’ fees
Specification	Glm, log link, negative binom. Family	Logit	glm, log link, gamma family	glm, log link, gamma family
PD	1.025(0.004)***	1.245(0.023)***	0.928 (0.011)***	0.86 (0.01)***
Age	1.002(0)***	0.995(0.001)***	1.004 (0)***	1.004 (0)***
Gender = m	0.957(0.003)***	1.18(0.02)***	1.107 (0.013)***	1.111 (0.013)***
PPO-contract	1.022(0.006)***	0.992(0.031)	0.944 (0.019).	0.941 (0.019)**
Telemedicine contract	0.973(0.005)***	0.994(0.026)	0.949 (0.017)***	0.942 (0.016)***
HMO contract	0.996(0.004)	1.123(0.022)***	0.935 (0.012)***	0.927 (0.012)***
High deductible	0.871(0.004)***	1.123(0.023)***	0.803 (0.011)***	0.801 (0.011)***
Cost in previous year (log)	1.049(0.001)***	0.936(0.004)***	1.171 (0.003)***	1.170 (0.003)***
Nursing home stay	0.989(0.008)	0.962(0.063)	0.960 (0.033)*	0.968 (0.033)
Outpatient hospital visits 1-5	1.184(0.004)***	0.846(0.019)***	1.151 (0.016)***	1.150 (0.016)***
Outpatient hospital visits > 5	1.182(0.009)***	0.81(0.045)***	1.198 (0.038)***	1.201 (0.037)***
Inpatient stay in hospital	1.066(0.006)***	0.782(0.032)***	1.081 (0.025)***	1.085 (0.024)***
Prescriptions psychiatrists	0.907(0.01) ***	1.125(0.065)*	1.814 (0.069)***	1.775 (0.066)***
Prescriptions cardio-/angiologists	1.002(0.034)	0.603(0.099)**	1.163 (0.125)*	1.132 (0.119)***
Prescriptions gynaeologists	1.091(0.01)***	0.311(0.016)***	1.283 (0.035)***	1.279 (0.034)***
Prescriptions other specialists	1.132(0.006)***	0.478(0.013)***	1.865 (0.032)***	1.821 (0.03)***
Agricultural municipalities	1.067(0.147)	0.638(0.377)	1.005 (0.415)	1.012 (0.408)
Affluent municipalities	1.067(0.147)	0.573(0.049)***	0.954 (0.058)	0.96 (0.057)
Industrial and tertiary municipalities	1.064(0.019)***	0.917(0.086)	0.949 (0.064)	0.95 (0.063)
Rural commuting municipalities	1.048(0.021)*	0.825(0.08)*	1.002 (0.069)	1.002 (0.068)
Periurban municipalities	1.016(0.021)	0.813(0.068)*	0.952 (0.057)	0.956 (0.056)
Suburban municipalities	1.019(0.018)	0.691(0.056)***	0.934 (0.055)	0.941 (0.054)
Touristic municipalities	1.052(0.018)**	0.883(0.195)	0.644 (0.095)	0.649 (0.094)**
Urban centers	0.961(0.044)	0.774(0.063)**	0.882 (0.052)	0.896 (0.052)
22 pharmaceutical cost groups	all coefficients strongly positive	most coefficients negative	all coefficients strongly positive	all coefficients strongly positive

Target variable per patient, first 6 months of year 2013. Exponential of coefficients [*exp*(^β)] displayed

Standard errors calculated by the delta method [*se*_*exp*(β)_ = *exp*(β)] ∗ *se*_β_, displayed in parentheses

Significance levels: ***p < 0.001, **p < 0.01, *p < 0.05

Source: Claims data from Helsana insurance company, own calculations

Hypothesis 2 is related to the share of generic drugs. The odds ratio of the PD variable is greater than 1 (see second column of Table 5), showing that PD patients receive more generic drugs than otherwise similar non-PD patients. The average marginal effect amounts to 3.6 percentage points. Our results replicate the finding of Rischatsch et al. [15] who also show that PD is associated with an increased use of generics.

The control variables reveal that older and sicker patients have a lower probability of using generic drugs, while males, and patients with high deductibles or managed-care type contracts have a higher probability of using them. There is a strong negative effect of using generics in affluent and (sub-)urban communities, which might indicate lower price sensitivity and/or a stronger preference for well-known brands among wealthier and more urban patients.

Results for pharmaceutical expenditures are shown in the third column of Table 5. PD patients are found to spend about 7 % less on pharmaceuticals relative to otherwise similar non-PD patients. Existing studies on the relation between PD and drug expenditures used either cantonal or physician-level data and found different results. The most recent study is by Kaiser et al. [13] who find a strongly positive association of PD with drug expenditures prescribed by specialists. The discrepancy might be related to the fact that roughly 60 % of the pharmaceutical spending was prescribed by GPs in the canton of Zurich in 2013 and this was not analysed by previous mentioned study [13]. However, the study performed by Burkhard et al. [14] suggests that physician dispensing leads to an increase of drug costs of 25 % for general practitioners and 15 % for specialists. The reason for the differing results may be attributed to the definition of a dispensing physician. The prior mentioned studies identify each physician by using the dispensing permission issued by the respective cantonal authorities. As not all physicians legally entitled to dispense actually use their permission in daily pratice, our definition utilized in this study may lead to different findings. In addition, results on the physician level may differ from results on the patient level if PD- and Non-PD-patients don’t show the same level of loyalty to their physicians. Using patient-level data, this study sheds new light on the issue by analyzing the association of PD on total drug expenditure per patient independently of the provider.

The control variables mostly have the expected signs. Pharmaceutical expenditures are higher for older patients, patients with hospital visits/stays, and patients predominately treated by specialists. Patients with high deductibles or managed-care type contracts have lower expected expenditures than other patients. The type-of-municipality variables turn out to be insignificant. After controlling for several indicators of utilization and choice of provider, the location of the patient seems not to be influential on pharmaceutical spending.

The fourth column of Table 5 shows the same estimation, but includes consultation fees for pharmacists in the calculation. These fees are paid to pharmacists for the dispensing of prescription drugs, and are part of the pharmaceutical bill. Physicians ‘consultation fees’ do not show on the pharmaceutical bill and will be analyzed in the next section. If consulting fees for pharmacists are added to pharmaceutical expenditure, PD patients have lower expenditures by even about 14 %, relative to comparable non-PD patients.

**Table 6:** Results of the multivariate regression analysis of the impact of physician dispensing (PD) on physician services

Target variable	Number of GP visits	Number of specialist visits	Probability of a physician visit	Physician expenditures if > 0
Specification	Glm, log link negative binom. fam.	Glm, log link negative binom. fam.	Probit	Glm, log link gamma family
PD	1.05 (0.008)***	1.065 (0.01)***	0.444 (0.014)***	1.02 (0.087)*
Age	1.002 (0)***	0.997 (0)***	−0.004 (0)***	1.003 (0)***
Gender = m	0.955 (0.006)***	0.826 (0.008)***	−0.12 (0.013)***	0.95 (0.008)***
PPO-contract	1.043 (0.012)***	1.001 (0.017)	0.072 (0.025)**	0.989 (0.014)
Telemedicine contract	1 (0.011)	1.055 (0.015)***	0.065 (0.02)**	1.022 (0.013)
HMO contract	1.032 (0.008)***	0.988 (0.011)	0.114 (0.016)***	0.981 (0.009)*
High deductible	0.882 (0.008)***	0.864 (0.01)***	−0.14 (0.015)***	0.921 (0.009)***
Cost in previous year (log)	1.06 (0.002)***	1.153 (0.003)***	0.649 (0.053)***	1.447 (0.022)***
Nursing home stay	1.334 (0.016)***	-	0.044 (0.003)***	1.068 (0.002)***
Outpatient hospital vistis 1-5	0.889 (0.016)***	1.559 (0.026)***	0.339 (0.063)***	0.777 (0.018)***
Outpatient hospital vistis >5	1.313 (0.01)***	1.365 (0.015)***	0.406 (0.021)***	1.313 (0.012)***
Inpatient stay in hospital	1.303 (0.021)***	1.259 (0.029)***	0.314 (0.055)***	1.238 (0.026)***
Prescriptions psychiatrists	0.409 (0.03)***	4.687 (0.362)***	0.238 (0.048)***	3.488 (0.091)***
Prescriptions cardio-/angiologists	0.278 (0.006)***	4.445 (0.091)***	−0.017 (0.102)	2.815 (0.221)***
Prescriptions gynaeologists	0.405 (0.01)***	10.156 (0.266)***	0.012 (0.029)	1.707 (0.033)***
Prescriptions other specialists	0.347 (0.004)***	5.582 (0.072)***	0.211 (0.02)***	2.25 (0.027)***
Agricultural municipalities	0.821 (0.198)	1.45 (0.554)	0.153 (0.411)	1.06 (0.309)
Industrial and tertiary municipalities	0.921 (0.221)	1.533 (0.583)	0.114 (0.075)	1.011 (0.048)
Rural commuting municipalities	0.903 (0.217)	1.579 (0.601)	0.071 (0.077)	1.054 (0.051)
Touristic municipalities	0.742 (0.189)	1.49 (0.587)	0.094 (0.154)	1.123 (0.117)
Affluent municipalities	0.959 (0.229)	1.948 (0.739)	0.228 (0.067)***	1.221 (0.052)***
Periurban municipalities	0.893 (0.213)	1.645 (0.624)	0.062 (0.066)	1.091 (0.046)*
Suburban municipalities	0.918 (0.219)	1.733 (0.657)	0.138 (0.064)*	1.123 (0.046)**
Urban centers	1.011 (0.242)	1.942 (0.736)	0.34 (0.064)***	1.249 (0.052)***
22 pharmaceutical cost groups	all coefficients strongly positive	all coefficients strongly positive	all coefficients strongly positive	all coefficients strongly positive

Target variable per patient, first 6 months of year 2013. Exponential of coefficients [*exp*(^β)] displayed in columns 1,2,4

Standard errors calculated by the delta method [ŝ_*exp*(β)_ = *exp*(β) ∗ ŝ_β_], displayed in parentheses

Significance levels: ***p < 0.001, **p < 0.01, *p < 0.05

Source: Claims data from Helsana insurance company, own calculations

**Table 7:** Results of the multivariate regression analysis of the impact of physician dispensing (PD) on total health care expenditures

Specification	Glm, log link gamma family
PD	0.989 (0.008)
Age	1.005 (0)***
Gender = m	0.977 (0.007)**
PPO-contract	0.953 (0.012)***
Telemedicine contract	0.996 (0.011)
HMO contract	0.965 (0.008)***
High deductible	0.87 (0.008)***
Nursing home stay	3.126 (0.069)***
Outpatient hospital visits 1-5	1.783 (0.016)***
Outpatient hospital visits >5	2.427 (0.048)***
Inpatient stay in hospital	4.003 (0.058)***
Cost in past year (log)	1.117 (0.002)***
Prescriptions psychiatrists	2.252 (0.054)***
Prescriptions cardio-/angiologists	1.614 (0.109)***
Prescriptions gynaeologists	1.488 (0.026)***
Prescriptions other specialists	1.78 (0.019)***
Agricultural municipalities	1.03 (0.268)
Affluent municipalities	1.074 (0.041)
Industrial and tertiary municipalities	0.977 (0.042)
Rural commuting municipalities	1.004 (0.044)
Periurban municipalities	1.025 (0.039)
Suburban municipalities	1.022 (0.038)
Touristic municipalities	0.946 (0.088)
Urban centers	1.079 (0.04)*
22 pharmaceutical cost groups	all coefficients strongly positive

Target variable per patient, first 6 months of year 2013. Exponential of coefficients [*exp*(^β)] displayed

Standard errors calculated by the delta method [ŝ_*exp*(β)_ = *exp*(β) ∗ ŝ_β_], displayed in parentheses

Significance levels: ***p < 0.001, **p < 0.01, *p < 0.05

Source: Claims data from Helsana insurance company, own calculations

**Table 8:** Coefficent and confidence interval for the relationship between PD and target variables

Variable	Coefficients	CI: 2.5 %	CI : 97.5 %
Total health care expenditures	0.989	0.974	1.004
Probability of a physician visit	0.444	0.416	0.472
Physician expenditures if > 0	1.02	1.003	1.037
Pharmaceutical exp. and pharmacists’ fees	0.86	0.84	0.88
Pharmaceutical expenditure	0.928	0.906	0.95
Number of GP visits	1.05	1.035	1.065
Number of specialist visits	1.065	1.045	1.086

Source: Claims data from Helsana insurance company, own calculations

### Physician services

In hypothesis 3 we stated that PD is likely to increase the supply of physician consultations. As shown in the first line of Table 6, this hypothesis cannot be rejected either. PD is associated with a higher number of visits to physicians. Contrary to our expectations, the association on GP care is not stronger than on specialist care. Calculating the marginal effects, we find that PD increases the expected number of GP visits by 0.122 visits per patient and the expected number of visits to specialists by 0.086 visits per patient. Kaiser et al. [13] also analyzed the impact of PD on visits to specialists and find similar effects (a plus of 0.067 or 0.081 visits per patient, depending on the specification).

In column three and four of 6, results are reported for physician expenditures, which are modelled by a two-part model. The influence of the PD variable is quite sizeable and strongly significant in the first part, meaning that PD increases the probability of using physician services. Once the use is initiated, expenditures are estimated to be 2 % higher for PD patients than for non-PD patients. Putting the two parts of the model together, we estimate PD patients on average to have 6.5 % higher expenditure (Swiss francs 50.80) for physician services than comparable non-PD patients. Busato et al. [12] analyzed the effect of PD on the expenditures for different physician groups and find PD to be associated with higher cost for some groups of specialists but not for primary care physicians, pediatric physicians, gynecologists and psychiatrists. However, their results are not directly comparable to ours as they use data from different cantons, and different cantons also have different prices for physician services.

### Total health care expenditures

The association of PD on total health care expenditure is not statistically significant (see Table 7). Two other articles addressing the question of PD and total health care expenditure are Reich et al. [9] who find a small positive effect of PD on expenditure and an older study by Vatter et al. [10] who find a small negative effect.

Still, it cannot be judged whether the enlargement of physician dispensing rights is beneficial for patients by analyzing expenditures only. To that regard, an analysis of treatment quality would be necessary, which is beyond the scope of this study. Recent evidence by Blozik et al. [17] showed that the probability of receiving a prescription of potentially inadequate medication among elderly patients is higher in the PD sector than in the non-PD sector. Finally, Table 8 illustrates and summarizes the coefficients and the according confidence intervals for the relationship between PD and the target variables.

### Robustness checks

We checked for the robustness of the results by applying several alternative calculations. First, we excluded the 5 % of patients with the highest drug expenditures from the calculations because rare, expensive drugs might be affected by PD in a different manner than ‘high volume’ drugs. Second, we excluded all patients who were treated by oncologist or rheumatologists. Several modern drugs in these specialties are administered directly to the patients, so most physicians in these specialties are dispensing some drugs (and earn their markups) due to the fact that the pharmacy is not an option. The results from these two tests remained within the confidence intervals of the results reported in section 5.

As a third test, we analyzed the impact of PD in two other cantons, Lucerne, which has a very high share of dispensing physicians and Argovia, where only a small portion of the physicians dispense. We expect that possible selection problems on the physician side are less accentuated between regions because many physicians have local ties. With respect to the associations of PD, the results we obtained in these two cantons had the same signs and significance levels and were of comparable size as those in Zurich. The conclusions remain the same.

Our conclusions are robust over several specifications tests. Still, we can not preclude the possibility that physicians who currently dispense might react differently to the incentives posed by dispensing than other physicians. If this bias exists, it most likely leads to an overestimation of the effects of physician dispensing in this study because physicians who are most likely to profit from dispensing select into regions and settings where regulations allows it.

Several strengths and limitations of our study have to be taken into account. The main strength is that the study was based on very comprehensive and practice-based health care claims data which covers a large population-based individuals. The study also has several limitations. First, our data did not embed information on clinical variables as well as further patient characteristics like employment, income, civil status and therefore potential confounding factors exist. Although our analyses were adjusted for numerous proxy variables indicating patients’ morbidity (costs in the previous year and pharmaceutical cost groups) and health insurance coverage, our information on the overall health status and socio-economic status are limited. Second, data may be underestimated since approximately 1.5 % of all claims invoices were not reimbursed by the health insurer and paid out-of-pocket by the individual patient. A third limitation of the study is that our estimates are not entirely representative of the general population in the canton of Zurich. However, this study is based on a widespread health care claims database including a large population from the health insurer with the highest market coverage in Zurich. Lastly, this study covers an observation period starting 8 months after legal frame changed in May 2012, which might not be sufficient enough to capture potential physicians’ behaviour change in terms of PD.

## Conclusions

Recent health care reforms in several countries have addressed the separation of labour between prescribers and sellers of drugs. For example, Taiwan and South Korea both passed reforms limiting the rights of physicians to dispense drugs, while on the other side of the pacific ocean, Canada or the US have recently expanded the rights of pharmacists to prescribe. In this context, it is important to analyze the differences between non-dispensing and dispensing prescribers of drugs.

This article sheds new light on the issue by analyzing data from the Swiss canton of Zurich. The canton of Zurich lends itself to analysis because many dispensing and non-dispensing physicians exist in an otherwise similar setting. From theoretical considerations and the existing literature, we stated three hypotheses. First, physician dispensing is expected to lead to higher drug quantities because physicians earn income from drug mark-ups. Second, physician dispensing is expected to be associated with a higher use of generic drugs because physicians are more aware of drug prices and can earn higher mark-ups with generic drugs (the latter is due to the Swiss regulation of drug prices). Third, with physicians’ earnings increased by dispensing, we expect an increase in the supply of ambulatory physician services.

Analyzing a large dataset of patient-level claims data, we could not reject any of the three hypotheses. Patients who buy their drugs mainly from physicians are found to use more active agents on average during the observation period, and to use generic drugs more frequently. In addition, physician dispensing was associated with an increased number of physician visits per patients. We also analyzed the effect of physician dispensing on expenditure and found it to be associated with lower drug expenditures per patient, but higher expenditures for ambulatory physician consultations. In terms of total health care expenditure, we did not find a significant association of physician dispensing.

This analysis offers insights for policy makers who are (re-)considering the separation between drug prescribing and dispensing, either by allowing physicians to dispense or pharmacists to prescribe certain drugs. In terms of total health care expenditures, we find no difference between the two systems, so we are doubtful that changing dispensing rights are a good measure to contain cost, at least in Switzerland. What we do find is an increased supply of ambulatory physicians services due to physician dispensing, which might or might not be desired politically. To know which system benefits patients more, an analysis of the impact of physician dispensing on medical outcomes would be necessary, which was beyond the scope of this study but is addressed in other recent research.

## Abbreviations

ATC, Anatomical Therapeutic Chemical; CHF, Swiss Francs; GP, general practitioner; HCE, health care expenditures; PD, physician dispensing; PID, provider-induced-demand; PIM, potentially inappropriate medications
